# β-arrestin-2 up-regulates toll-like receptor 2 signaling and inhibits apoptosis in human endometrial cancer heterotransplants in nude mice

**DOI:** 10.1186/s12885-019-6254-4

**Published:** 2019-11-01

**Authors:** Fanling Hong, Yujun Zhang, Wenjin Cheng, Xiuli Sun, Jianliu Wang

**Affiliations:** 1 Department of Obstetrics and Gynecology, Peking University People’s Hospital, No.11 Xizhimen South Street, Xicheng Dist, Beijing, 100044 China; 2 The Clinical Institute of Molecular Biology & Central Lab, Peking University People’s Hospital, No.11 Xizhimen South Street, Xicheng Dist, Beijing, 100044 China

**Keywords:** Endometrial carcinoma, Toll-like receptor 2, β-arrestin-2, Apoptosis, Cell proliferation, Invasion

## Abstract

**Background:**

β-arrestin-2(Arr2) functions as an anti-apoptotic factor and affects cell proliferation, but its downstream molecular pathway in endometrial carcinoma (EC) is still unclear. This study aimed to investigate the effects of the stable overexpression of Arr2 on the proliferation and apoptosis of human EC heterotransplants and the expression of associated molecules, including Toll-like receptor 2(TLR2), serine-threonine kinase Akt (Akt), glycogen synthase kinase-3β(GSK3β) and some typical inflammatory cytokines such as NF-κB p56, TNF-α and IL-6 & IL-8.

**Methods:**

Human EC cell line Ishikawa, stably transfected with Arr2 full-length plasmid, was injected subcutaneously into nude mice. They were treated with 0, 10, 20 mg/kg paclitaxel and the volume and weight of the tumor tissue were measured and calculated. The necrotic index were assessed by H&E staining and microscopic observation. The levels of caspase-3, caspase-9, TLR2, NF-κB p56, Akt, GSK3β were measured by western blot, and the levels of TNF-α, IL-6, IL-8 were measured by real-time PCR.

**Results:**

We found that Arr2 overexpression promoted the growth of human EC heterotransplants. Arr2 attenuated the promotion of caspase-3 and caspase-9 by paclitaxel and mediated the increase of TLR2 and several inflammatory cytokines. The levels of Akt and GSK3β were not affected.

**Conclusion:**

Arr2 overexpression was associated with the increase of TLR2 and several inflammatory factors, meanwhile inhibited paclitaxel-induced anti-tumor effect on human EC heterotransplants.

## Background

EC is the fourth most common gynecologic malignancy in developed countries [18] and is now gaining increasing prevalence even in historically lower risk regions such as Asia [11]. Treatment for EC includes a combination of surgery, radiotherapy and chemotherapy. Doxorubicin, platinum drugs and paclitaxel are known for their activity against EC [19], and are commonly used in the chemotherapy regimen. Unfortunately, cancer resistance to these drugs could be a critical issue affecting survival, calling for further studies on EC pathogenesis and drug resistance.

Arr2, a member of the arrestin family, was proved to play a critical role in the anti-apoptotic pathway [12, 21, 22] while TLR2, a member of toll-like receptors family, played an important role in innate inflammatory response [1], possibly affecting the progress and metastasis of cancer [10]. Several studies showed the negative regulating effect of Arr2 on TLR2 signaling by interacting with mediators of the signaling pathway [15, 28], but there also were studies showing opposite results [13], including some of our in vitro data waiting to be published.

Some types of EC were associated with higher expression of genes involved in immune responses [8]. Among these, we focused on several pro-inflammatory cytokines such as NF-κB, TNF-α, IL-6 & IL-8, which was proved to have a positive effect on EC pathogenesis [6, 7, 24]. Furthermore, it was proved that Arr2 was required for NF-κB activation and IL-6 expression [25].

Several evidence indicated the modulating effect of Arr2 on Akt [3, 4, 21, 28], which was considered to attenuate cell apoptosis and promote cell survival. Likewise, GSK3β, one of the two isoforms of a serine/threonine kinase, had a regulatory impact on cell apoptosis [5, 9, 17]. Phosphorylation of GSK3β on the inactivating residue serine 9 by Akt led to GSK3β inactivation [17], which resulted in the reduce of apoptosis [30]. There was another evidence showing that apoptotic cascade mediated by GSK3β was attenuated by Arr 2[14]. Our previous study focusing on EC cells showed a similar result [26].

In the current study, we used paclitaxel to induce apoptosis of cancer heterotransplants in vivo, so as to reveal the function of Arr2 on tumor growth and associated molecular changes including the conflicting TLR2. Hopefully it will provide a better understanding of EC pathogenesis and drug resistance, which will eventually guide treatment.

## Methods

### Cell culture, transfection, and treatment

Human EC Ishikawa cell line (Beijing Union Cell Bank, China) were maintained in a basal medium (Dulbecco’s modified Eagle’s medium; Invitrogen, Carlsbad, CA, USA) supplemented with 10% FBS (Invitrogen, Carlsbad, CA, USA) in a 37°C humidified incubator with 5% CO_2_. The Arr2 full-length vector and the GFP vector were generous gifts from Dr. Gang Pei (Shanghai Institutes for Biological Sciences, China) [26].

Ishikawa cells (1 × 10^5^) were seeded on 24-well plates for 48 h before transfection. Transfection was performed with 1 μg of either Arr2 full-length vector or GFP vector using Lipofectamine 2000 (Invitrogen Corporation, Carlsbad, CA, USA), according to the manufacturer’s instructions. Forty-eight hours later, the medium was replaced with the basal medium containing 1 μg of G418. After screening by G418 for 2 weeks, single clone was selected and seeded in 35-mm dishes. Stable transfection was verified by western blot, namely, the Ishikawa/Arr2^+^ cell line and the Ishikawa/GFP cell line.

### Animals

Female BALB/c nude mice 6 to 8 weeks old were purchased from Shanghai Ling Chuang Biotechnology. Mice were housed under pathogen-free conditions with 12 h light/12 h dark, temperature of 20–25 °C and humidity of 40–70%. Mice had free access to complete nutritional palletized feedings and drinking water. Mice were subcutaneously injected in their right armpit with 0.1 ml cell suspension of 1 × 10^7^/ml in vitro cultured Ishikawa cells to initiate tumor growth. In vivo experiments in our study were performed according to the Institutional Animal Care and Use Committee (IACUC) guidelines. All procedures were approved by the Peking University People’s Hospital Committee on Animal Care.

### In vivo studies

Mice were randomly divided into two groups with 15 mice in each group. The control group were injected with Ishikawa/GFP cells and the Arr2+ group were injected with Ishikawa/Arr2^+^ cells. On Day14 when the size of tumor reached 75-100 mm^3^, each group of mice were randomly divided into three subgroups with 5 mice in each. On Day14 and Day21, the three subgroups of mice were intraperitoneally injected with normal saline (NS), 10 mg/kg paclitaxel and 20 mg/kg paclitaxel respectively. The xenografted tumor size was measured using vernier caliper every 2 to 3 days. Tumor volume (TV) was calculated by the formula 1/2 × A × B^2^, where A is the long diameter, B is the short diameter. Relative tumor volume (RTV) was calculated by V_t_/V_0_ where V_0_ is the TV right before paclitaxel was first given, and V_t_ was the TV measure each time after that. To evaluate anti-tumor activity, relative tumor growth ratio T/C(%) and anti-tumor activity index were calculated by formula shown as follow:

$$ T/C\left(\%\right)=\frac{T_{RTV}}{C_{RTV}}\times 100\% $$

T_RTV:_ RTV in treatment group, C_RTV:_RTV in control group.

All of the animals were euthanized on Day28 by CO_2_ inhalation and the xenograft were taken for the measurement of tumor weight. Tumor tissues were taken for further assessment of cell necrosis and expression of different molecules.

### Hematoxylin- and-eosin (H&E) staining

All tissues were fixed in 4% neutralised formaldehyde, embedded in paraffin, cut into 4-Ìm sections and stained by hematoxylin- and-eosin (H&E) to confirm their histological diagnosis and other microscopic characteristics. Assessment criteria included tumor cell morphology, necrosis, angiogenesis, inflammatory cell infiltration and fibrous tissue formation. Necrotic scores from 0 to 4 were given according to the percentage of necrotic area. 12%~ 25% was given 1 point, 25%~ 50% was given 2 points, 50%~ 75% was given 3 points. The tissue with necrotic area above 75% were given 4 points and those less than 12% were given 0.5 points. If there wasn’t any necrosis in the observed area, 0 point was given. Three different visual areas were chosen randomly in each section and their average was used in the analysis.

### Western blotting

Tumor tissues were lysed in RIPA buffer (Kaiji Biotech, Nanjing, China) with protease inhibitors (Amresco, Solon, OH, USA). After centrifugation at 14,000 rpm for 15 min, the supernatants were collected. Proteins were quantified using the BCA assay. Protein samples (20–30 μg) were resolved by 10% SDS-PAGE under reducing conditions and subjected to western blot analysis. After protein transfer to a PVDF membrane (Amersham, GE Healthcare, Waukesha, WI, USA), the membrane was blocked overnight at 4 °C using 5% BSA blocking buffer. The antibodies used for western blotting were 1:500 human monoclonal anti-β-arrestin-2 (Abcam, Cambridge, MA, USA, Ab54790), 1:1000 human monoclonal anti-TLR2 (Abcam, Cambridge, MA, USA, Ab24192), and 1:500 human monoclonal AKT (Kaiji Biotech, Nanjing, China, KG21054), GSK3β(Kaiji Biotech, Nanjing, China, KG21002), NFkBp65(Kaiji Biotech, Nanjing, China, KGYM0474), caspase3(Kaiji Biotech, Nanjing, China, KG22205) and caspase9(Kaiji Biotech, Nanjing, China, KG22222) antibodies. The secondary antibody was 1:2000 horseradish peroxidase-conjugated antibody (Kaiji Biotech, Nanjing, China). Western blots were developed by ECL (Pierce Chemical, Dallas, TX, USA). The bands were quantified using the Quantity One V4.52 software (Bio-Rad, Hercules, CA, USA) and were normalized with the density of GADPH bands.

### Immunofluorescence microscopy

Tumor tissues were fixed and incubated in a blocking buffer (0.5% Triton X-100, 1% BSA-PBS) for 1 h at room temperature followed by incubation with 1:200 human monoclonal anti-β-arrestin-2 (Abcam, Cambridge, MA, USA, Ab54790), 1:200 human monoclonal anti-TLR2 (Abcam, Cambridge, MA, USA, Ab24192) at 4 °C overnight. They were then incubated with antibody conjugated with fluorphores at room temperature for 1 h, next to 5 mg/ml DAPI for 10 min for nuclear staining. The fluorescence signals were observed under a fluorescence microscope (Olympus, Japan).

### Real-time PCR

Total RNAs were extracted from tissues with TRIzol (Invitrogen) according to the manufacturer’s instructions. Reverse transcription of 2 μg of the purified RNA was performed using RevertAid™First Strand cDNA Synthesis Kit (Thermo Fisher, USA). Then quantification of the genes was performed by real-time PCR, using ABI Step one plus Real time-PCR system with Real time PCR Master Mix (SYBR Green) (TOYOBO, Japan). Expression values were normalized to those obtained with control GAPDH. The primer pairs are as follows:

GAPDH, Sense 5′-TATGTCGTGGAGTCTACTGGT-3′, Anti-sense 5′-GAGTTGTCATATTTCTCGTGG -3′;

IL6, Sense 5′-CAATGGCAATTCTGATTGTATG-3′, Anti-sense 5′-AGGACTCTGGCTTTGTCTTTC-3′.

IL8, Sense 5′-TGTTGAGCATGAAAAGCCTCTAT-3′, Anti-sense, 5′-AGGTCTCCCGAATTGGAAAGG-3′.

TNF-α, Sense 5′-CCTGTAGCCCACGTCGTAG-3′, Anti-sense, 5′-GGGAGTAGACAAGGTACAACCC-3′.

### Statistical analysis

All statistical analyses were conducted using SPSS 25 (IBM, Armonk, NY, USA). Data was expressed as mean ± standard deviation (SD) from at least three independent experiments. The differences among groups were assessed using one-way analysis of variance (ANOVA) with the Bonferroni’s post hoc test. Two-sided *P*-values < 0.05 were considered statistically significant, while *P*-value < 0.01 were considered very significant.

## Results

### Arr2 promoted the growth of human EC heterotransplants

**Fig. 1:**
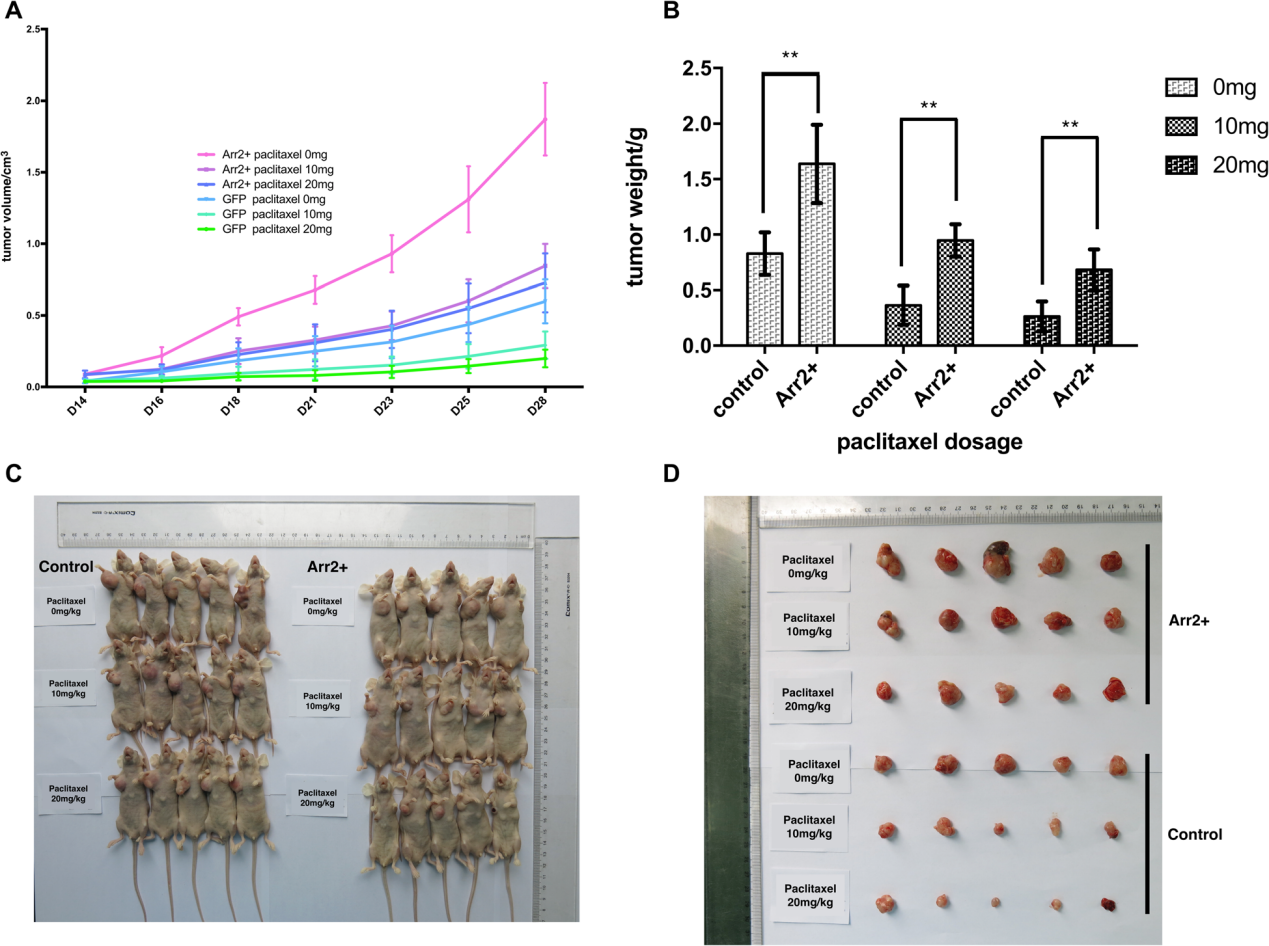
Tumor volume and tumor weight after paclitaxel treatment (*P < 0.05, ** *p* < 0.01). **a** Tumor volume in each group was monitored from D14 to D28. **b** Tumor weight was measured after sacrificing on D28. In both control and Arr2+ mice, 10 mg(*P* = 0.002, *P* = 0.000, respectively) and 20 mg(*P* = 0.000, both) paclitaxel treatment led to a significant lower level of tumor weight comparing to 0 mg treatment. **c** Mice of both groups with tumor heterotransplants in the right ampit after sacrificing on D28. **d** Tumor heterotransplants of both groups after sacrificing on D28

We investigated the change of tumor volume and weight in both control and Arr2^+^ group without paclitaxel treatment. From Day14 to Day28, tumor volume in both groups were growing steadily. Tumor volume in Arr2^+^ group was significantly larger than that in the control group, and the gap between two groups gradually increased (Fig. 1a). RTV was 15.347 ± 3.695 in control group and 21.466 ± 4.914 in Arr2+ group on Day28 (Table 1). And Arr2+ mice had significantly larger weight than control in each dosage group (Fig. 1b). These results altogether showed the promotion effect of Arr2 on tumor growth.

**Table 1:**
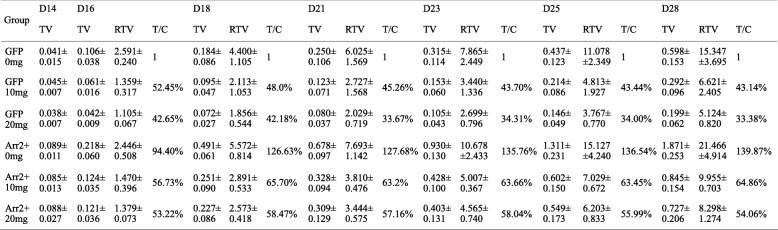
TV, RTV, T/C in different groups from D14 to D28

### Arr2 limited tumor tissue necrosis

**Fig. 2:**
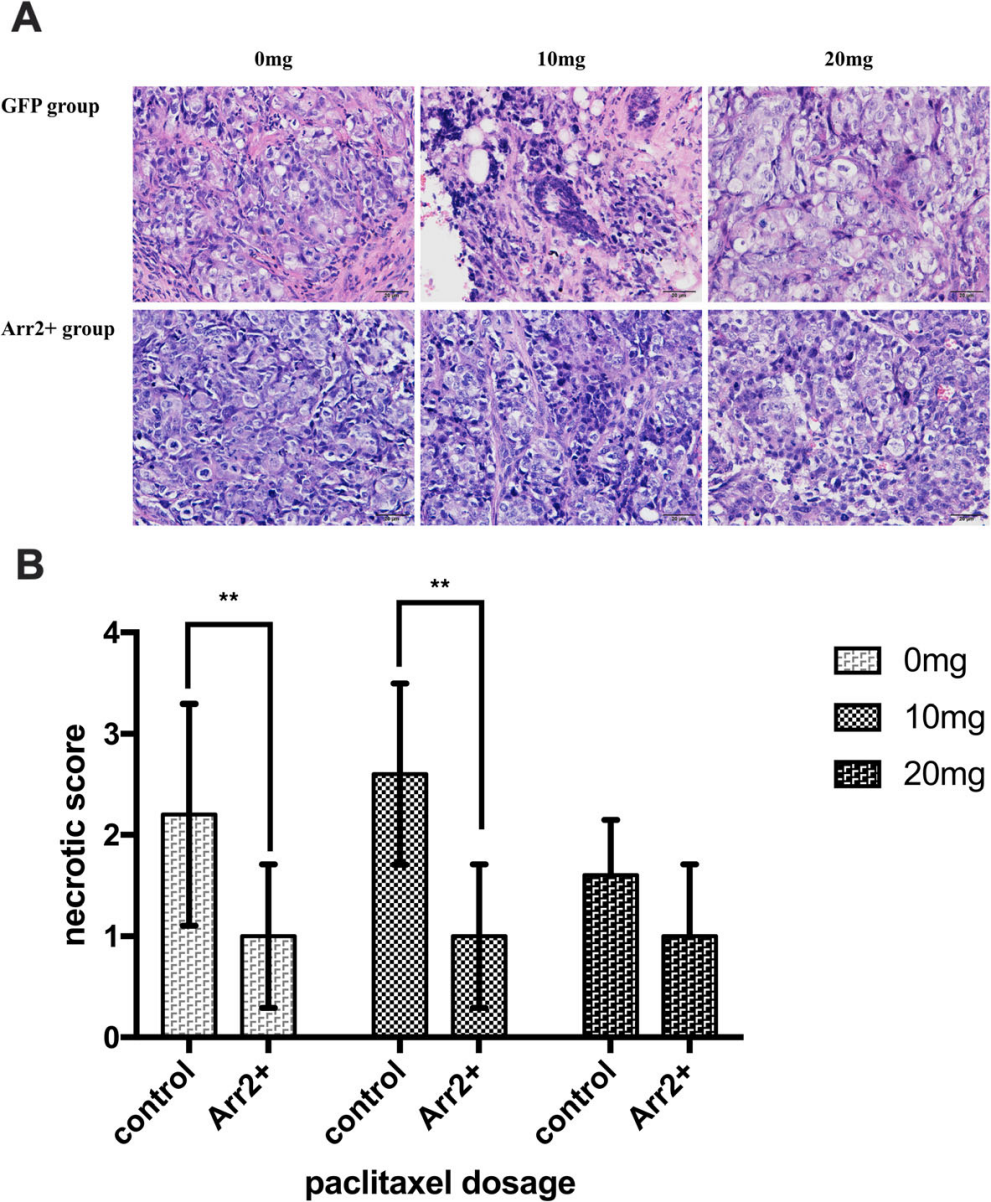
The necrosis was detected by H&E staining and different scores were given according to the percentage of necrotic area. **a** Representative images of sections of tumor tissue from each group with different dosage of paclitaxel treatment. **b** The necrotic score of different groups were shown. (** *P* < 0.05)

**Fig. 3:**
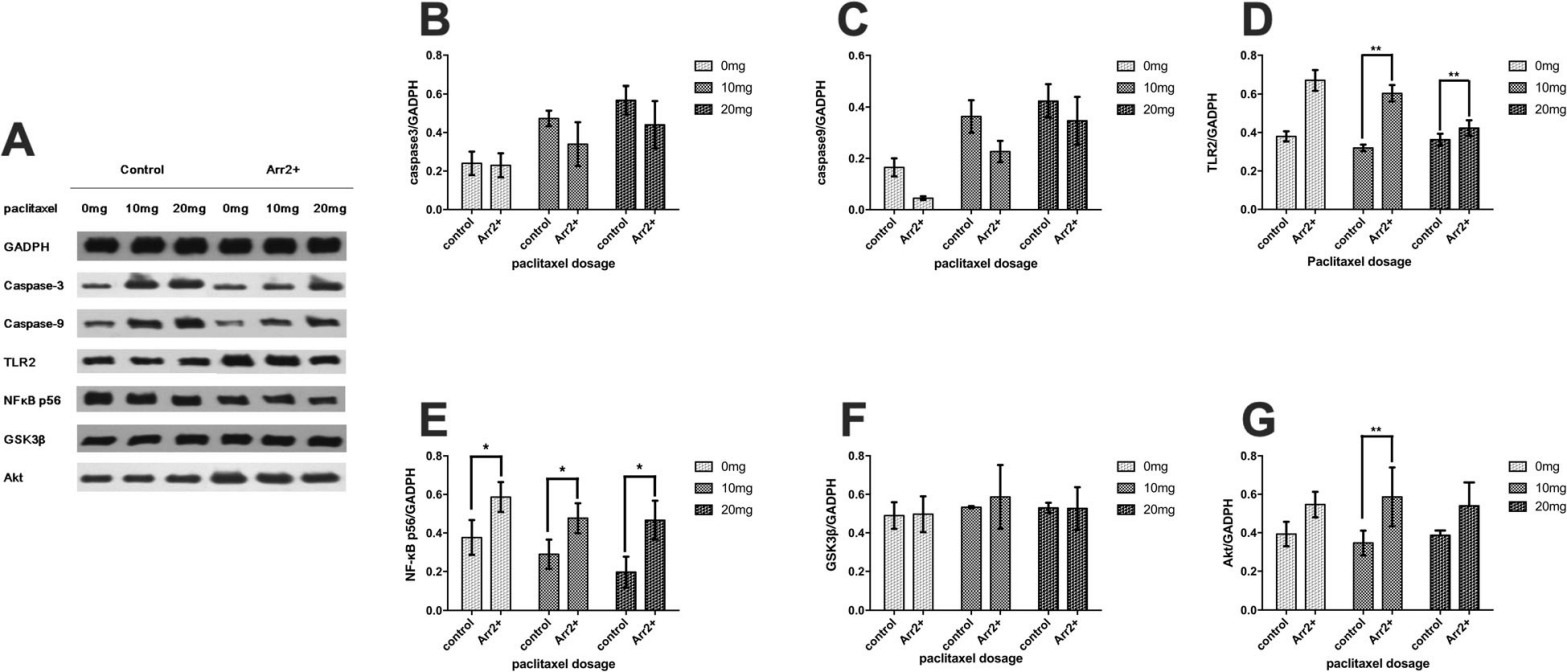
The expression of caspase-3, caspase-9, TLR2, NF-κB p56, GSK3β, Akt was assessed by western blot (* *P* < 0.05, ** *P* < 0.01). **a** The expression of the molecules, normalized by GADPH. **b**~**c** The expression of caspase-3 and caspase-9 in Arr2+ mice and control with different dosage of paclitaxel treatment was shown In Arr2+ mice, 20 mg paclitaxel group has significantly higher level of caspase-9 expression than 0 mg group(*P* = 0.003). In the control, significant effect can be seen between 20 mg and 0 mg group in caspase-3 (*P* = 0.007) and between both 0 mg, 10 mg (*P* = 0.012) and 0 mg, 20 mg group (=0.002) in caspase-9. **d** The expression of TLR2 in each group was shown. Both 20 mg(*P* = 0.000) and 10 mg(*P* = 0.003) paclitaxel group show significance comparing to 0 mg group in Arr2+ mice, which is not shown in control group. (**e**~**g**) The expression of NF-κB, GSK3β and Akt in each group was shown. There was no significance between each treatment group in Arr2+ and control mice, except 20 mg and 0 mg group in control mice (*P* = 0.022)

H&E staining and microscopic assessment of tumor tissue necrosis showed that the necrotic score of each Arr2+ group with 0 mg and 10 mg paclitaxel treatment was significantly lower than its counterpart in control group (Fig. 2). We further assessed the expression of apoptotic marker caspase-3 and caspase-9 in each group. Neither of the markers showed significant difference between Arr2+ group and control group, regardless of paclitaxel dosage (Fig. 3b, c).

### Arr2 was associated with higher expression of TLR2 and several inflammatory factors

**Fig. 4:**
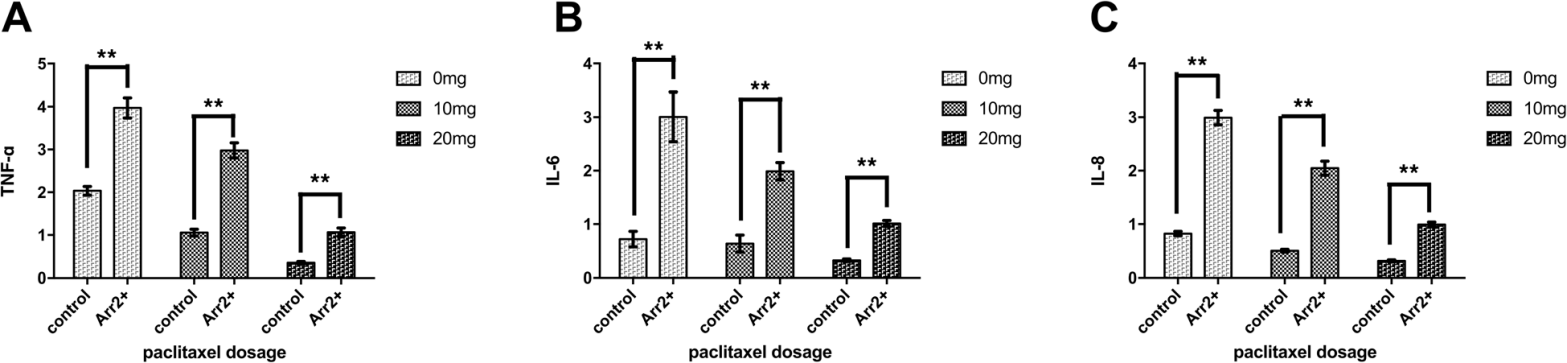
**a**~**c** The expression of TNF-α, IL-6, IL-8 in Arr2+ mice and control with different dosage of paclitaxel treatment was assessed with real-time PCR, and was measured by 2-ΔΔCT(** *P* < 0.01). For all three factors, *P* = 0.000 between each treatment group for both control and Arr2+ mice except those between 20 mg and 10 mg (*P* = 0.050), and between 10 mg and 0 mg (*P* = 0.051) in control for the measurement of IL-6

We tested the expression of TLR2 and some downstream factors in each group. In Arr2+ group, there was significantly higher level of TLR2 expression in 10 mg and 20 mg treatment group comparing to the corresponding control (Fig. 3d). The levels of NF-κB, TNF-α, IL-6 & IL-8 in Arr2+ group were significantly higher than their corresponding control with each dosage of paclitaxel treatment (Figs. 3e, and 4). But neither Akt nor GSK3β in Arr2+ group showed significant difference with control (Fig. 3f, g).

### Arr2 was associated with higher resistance of human EC heterotransplants to paclitaxel

We compared the sensitivity of EC heterotransplants to different dosage of paclitaxel between the two groups. Tumor volume was significantly lower in control groups and was even lower when given higher dosage of paclitaxel (Fig. 1a). T/C on Day28 were 64.86 and 54.06% respectively in Arr2 group when given 10 mg/kg and 20 mg/kg paclitaxel, while the corresponding number being 43.14 and 33.38% in control groups (Table 1). Tumor weight on Day28 was significantly smaller with increased dosage of paclitaxel (Fig. 1b). These data suggested that Arr2+ heterotransplants were more resistant to paclitaxel treatment.

### Arr2 attenuated caspase-3 and caspase-9 promotion following paclitaxel treatment

There wasn’t any change in the necrotic score with increasing dosage of paclitaxel treatment. On the other hand, there was a significant increase in caspase-3 with larger doses of paclitaxel treatment in control group but this effect was not seen in Arr2+ group (Fig. 3b, c), which indicated that larger doses of paclitaxel could be inducing tumor cell apoptosis, but this effect was attenuated by overexpression of Arr2.

### Arr2 mediated the increase of TLR2 following paclitaxel treatment

**Fig. 5:**
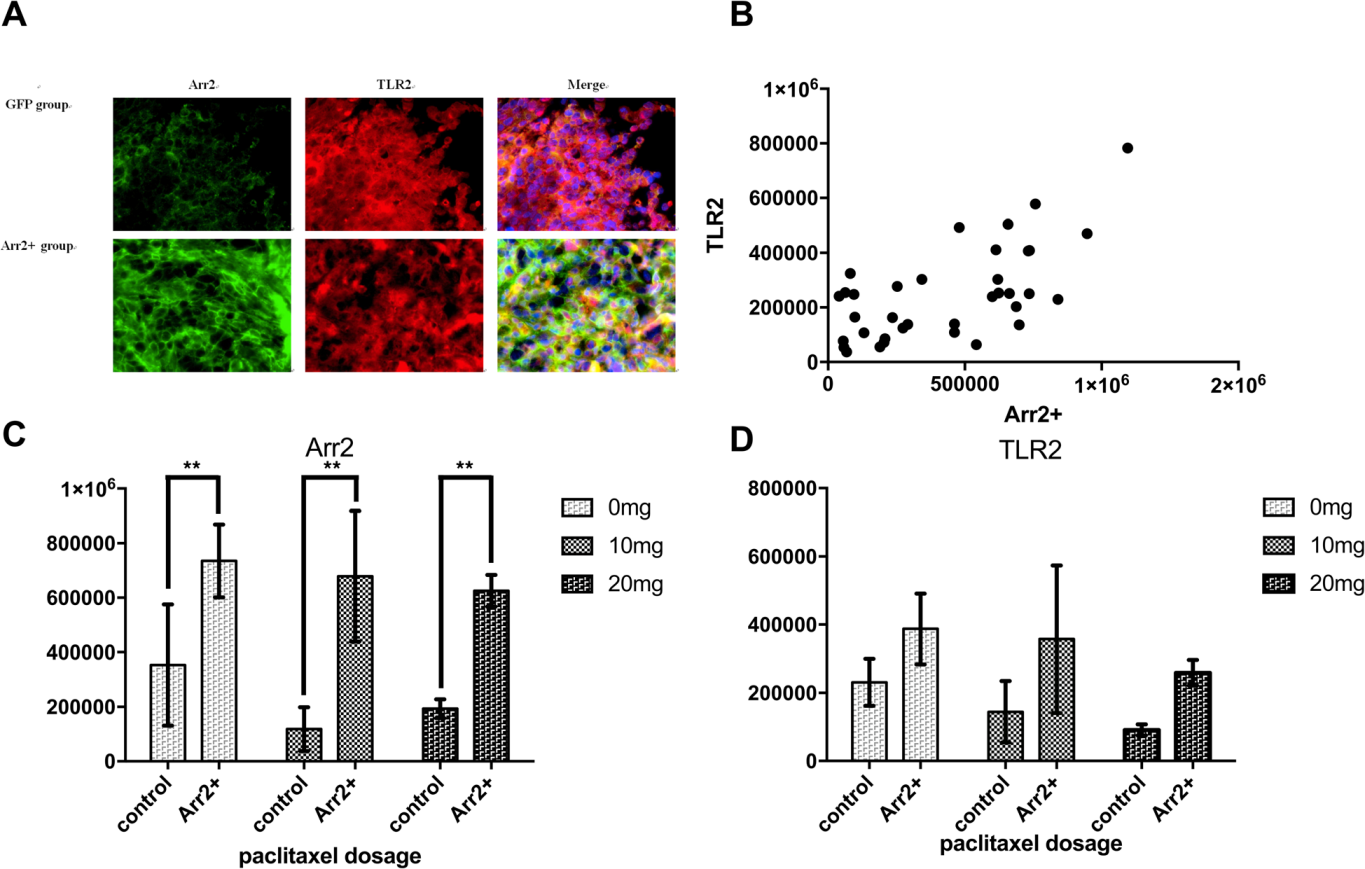
The expression of TLR2 and Arr2 in Arr2+ mice and control was detected by immunofluorescence. **a** Representative images of frozen sections of tumor tissue from both groups without paclitaxel treatment, immunostained with anti-TLR2(red) and anti-Arr2 (green). **b** The IOD of TLR2 correlates with the IOD of Arr2+ in all samples. **c**~**d** the IOD of Arr2+ and TLR2 in each group

A significant decrease in TLR2 was found in Arr2+ group when treated with 10 mg and 20 mg paclitaxel comparing with no treatment. On the other hand, in control group neither dosage of paclitaxel treatment led to a significant change comparing to the subgroup with no treatment at all (Fig. 3d and 5). These results suggested that the effect of Arr2 on TLR2 could be attenuated by larger dosage of paclitaxel treatment. In the meantime, there was a significant decrease in TNF-α, IL-6 & IL-8 with rising paclitaxel dosage in both control and Arr2+ group while little change was found in the level of NF-κΒ, Akt or GSK3β following the increase of dosage (Fig. 3e, f, g and 4).

## Discussion

Arr2, as a member of arrestin family, is widely expressed in many organs and tissue. It has been attracting people’s attention by its negative regulative effect on cell apoptosis, which might contribute to tumor pathogenesis [21, 22]. Results from the current in vivo study showed the promoting effect of Arr2 on the growth of human EC heterotransplants, as well as its contribution to their paclitaxel-resistance. In addition, the change of apoptotic markers caspase-3 and caspase-9 along with the results from the pathological assessment of necrosis further confirmed its positive regulatory effect on tumor pathogenesis in the molecular level.

In order to further understand the molecular mechanism of Arr2 on apoptosis, several potential downstream factors have been investigated, including TLR2. As a member of toll-like receptors family, TLR2 has been shown to mediate cancer metastasis by generating an inflammatory microenvironment hospitable for metastatic growth [10]. Unfortunately, when it comes to the relationship of Arr2 and TLRs, much confusion has been raised by conflicting results. While previous studies revealed the negative-regulating effect of Arr2 on TLRs [14, 28], recent ones focusing on TLR2 have brought up opposite opinions [13, 29]. A recent study confirming downregulation of TLR4 and upregulation of TLR2 in colorectal carcinomas [20] may partly explain these conflicts by indicating contrast effects of different TLRs on tumor pathogenesis. Our in vitro data (waiting to be published) together with the in vivo data from the current study showed that Arr2 was associated with the increase of TLR2, explaining the possible mechanism of its positive regulation on tumor pathogenesis.

Furthermore, interesting findings have indicated a possible association between TLR2 and the release of inflammatory cytokines, which contribute to a permissive microenvironment for tumor progression [10, 23]. In the present study, Arr2+ group showed significant increase of different pro-inflammatory cytokines comparing to control. Through what have been discussed about association between Arr2 and TLR2 previously, we may come to the conclusion that Arr2 up-regulates TLR2 signaling followed by the activation of several inflammatory cytokines release including IL-6, IL-8 and TNF-α, which induce tumor cell proliferation and inhibit apoptosis. In addition, NF-κB, known to be promoting inflammatory cytokine expression [2], and was found to be critical in TLR and Akt-mediated signaling [24, 27], also provided significant evidence in the current study. Notably, both TLR2 and inflammatory cytokines’ promotion were attenuated by larger dosage of paclitaxel treatment, which suggested the dose-dependent effect of paclitaxel and a possible treatment solution.

Akt and GSK3β, as two of the downstream molecules of Arr2 signaling pathway, has also been attracting people’s attention. In fact, evidence has shown that activated Akt leads to the inhibition of GSK3β by phosphorylation on the inactivating serine 9[9]. Interestingly, GSK3β has paradoxical pro-and anti-apoptotic actions, promoting cell death caused by the mitochondrial intrinsic apoptotic pathway, while inhibiting the death receptor-mediated extrinsic apoptotic signaling pathway [5]. It may lead to contradictary results when it comes to the effect of Arr2 on both molecules, unless taking into acount the apoptotic pathway in certain studies. An in vivo study on liver injury demonstrated that Arr2 deficiency enhances previously described Akt/GSK3β pathway to promote survival [30]. On the contrary, Our previous study in vitro showed that Arr2 attenuated resveratrol reduced level of p-Akt and p-GSK3 β[26], which is consistent with its anti-apoptotic theory. However, the current study didn’t show significant association between Arr2 and levels of Akt and GSK3β following paclitaxel treatment. These results might imply a different apoptotic pathway induced by paclitaxel. Future studies should take into account different apoptotic pathways in cell or animal models in order to have a clearer inspection of Arr2 and certain molecular pathways. More interestingly, TLR2’s promoting effect on colorectal cancer cell proliferation was shown by a recent study to be dependent on PI3K/Akt and NF-κB signaling pathways, which is inspiring for future studies on more detailed molecular pathways [16].

From various studies stated above, we may indicate that the contribution of Arr2 on tumor pathogenesis was associated with several apoptotic and inflammatory pathways, which was also our focus. The current study, exploring the possible role of Arr2 on tumor pathogenesis and its underlining mechanism in vivo, was, however, still limited. More comprehensive studies are needed to unveil a clearer pathway of Arr2 regulation on tumor progression.

## Conclusion

Arr2 overexpression inhibited paclitaxel-induced anti-tumor effect on human EC heterotransplants. It was also associated with the increase of TLR2 and several inflammatory factors. Therefore, Arr2 and TLR2 can be considered as potential targets for EC treatment.

## Data Availability

The datasets generated during and/or analysed during the current study are available from the corresponding author on reasonable request.

## Abbreviations

Arr2: β-arrestin-2
EC: endometrial carcinoma
TLR2: toll-like receptor 2
